# Consumption of dairy products and CVD risk: results from the French prospective cohort NutriNet-Santé – CORRIGENDUM

**DOI:** 10.1017/S0007114522000812

**Published:** 2022-09-28

**Authors:** Laury Sellem, Bernard Srour, Kim G. Jackson, Serge Hercberg, Pilar Galan, Emmanuelle Kesse-Guyot, Chantal Julia, Léopold Fezeu, Mélanie Deschasaux-Tanguy, Julie Lovegrove, Mathilde Touvier

British Journal of Nutrition, Volume 127, Issue 5, 14 March 2022, pp. 752 – 762

**Details of correction: Amend author name detail**

**Existing text:**

Julie Lovegrove

**Corrected text should read:**

Julie A. Lovegrove

